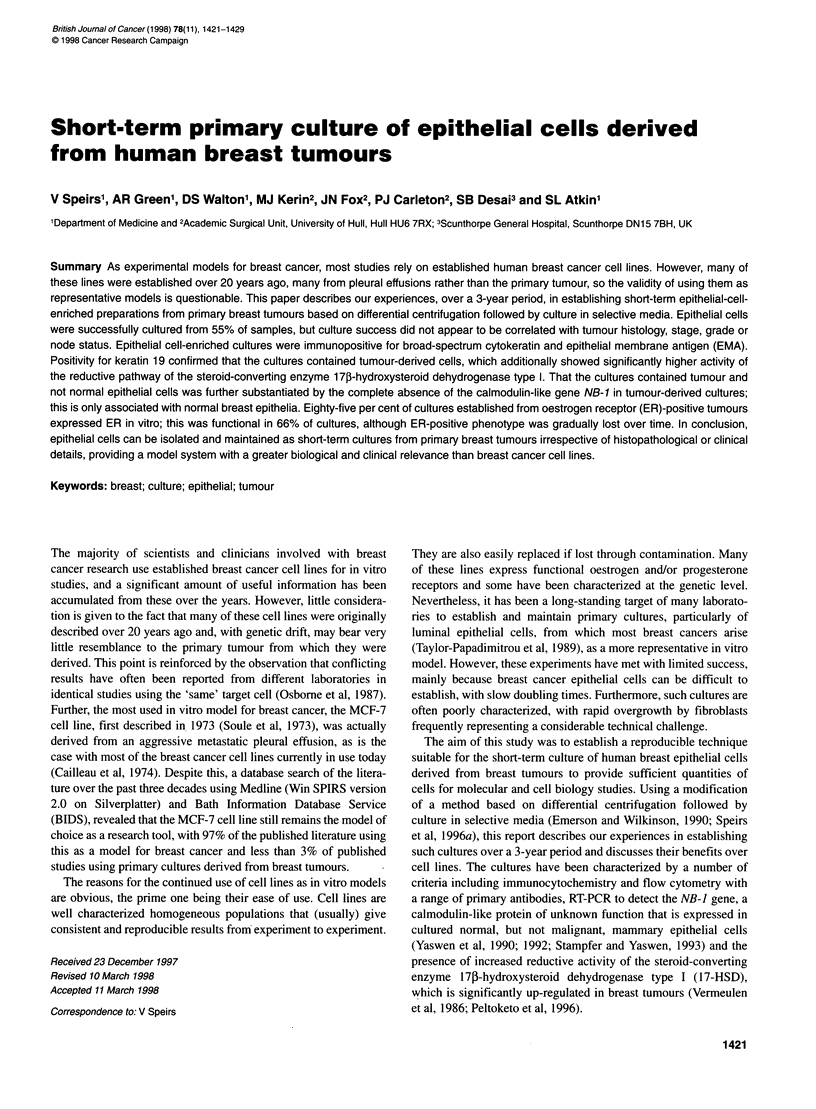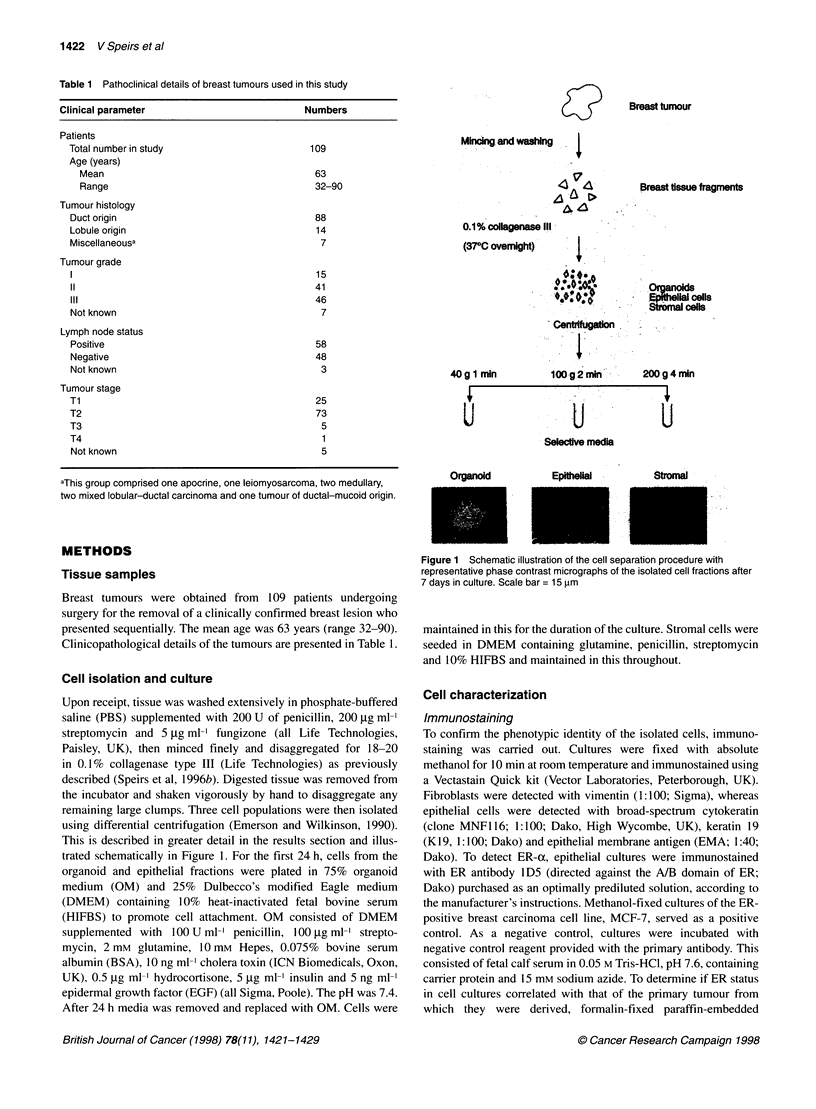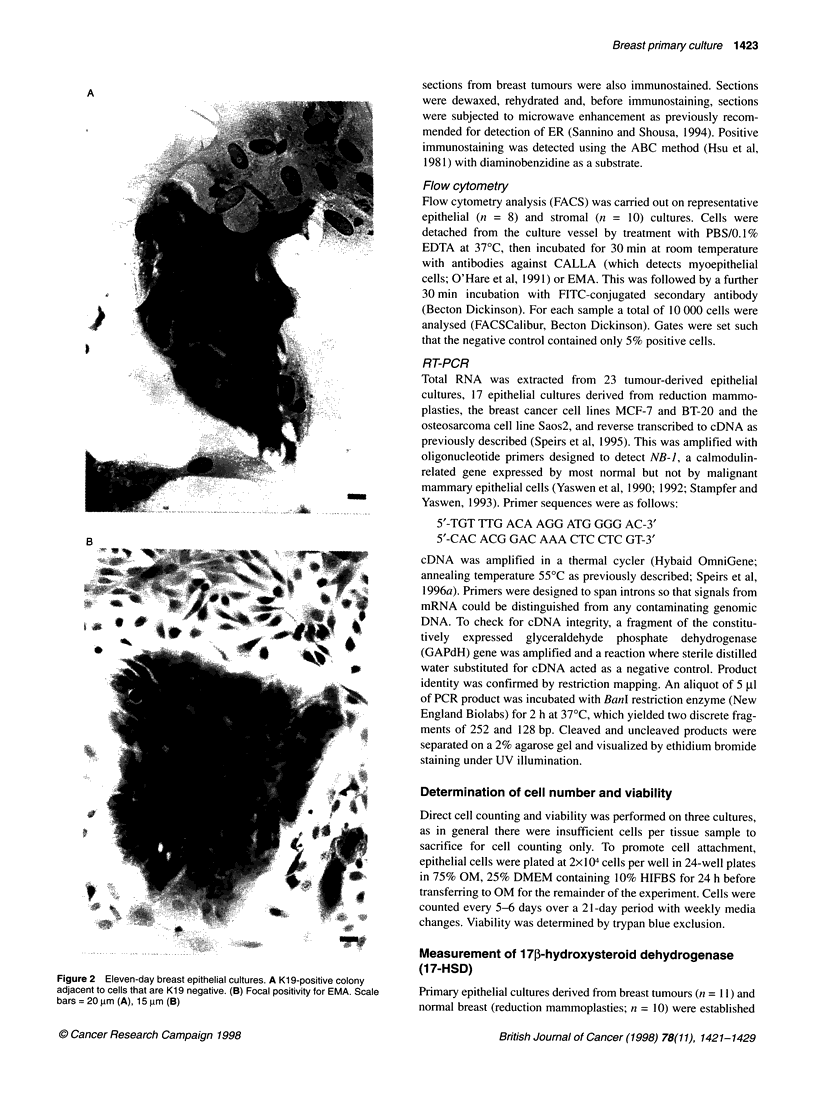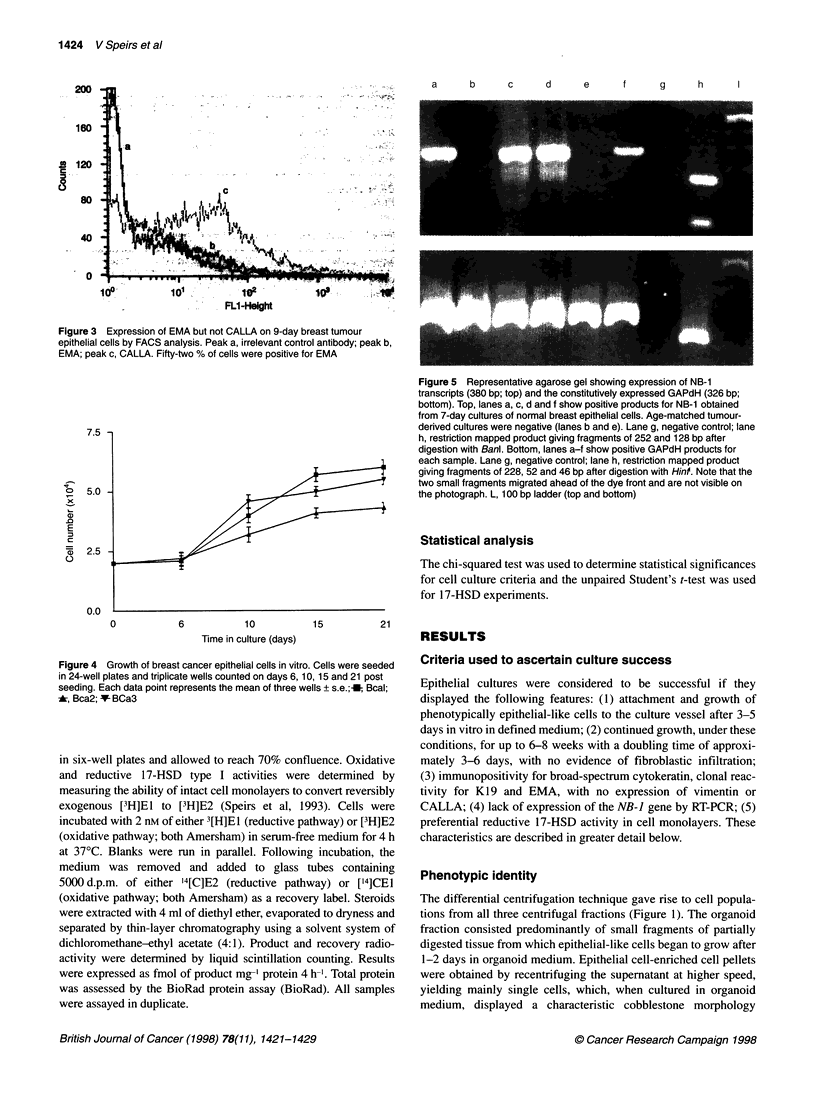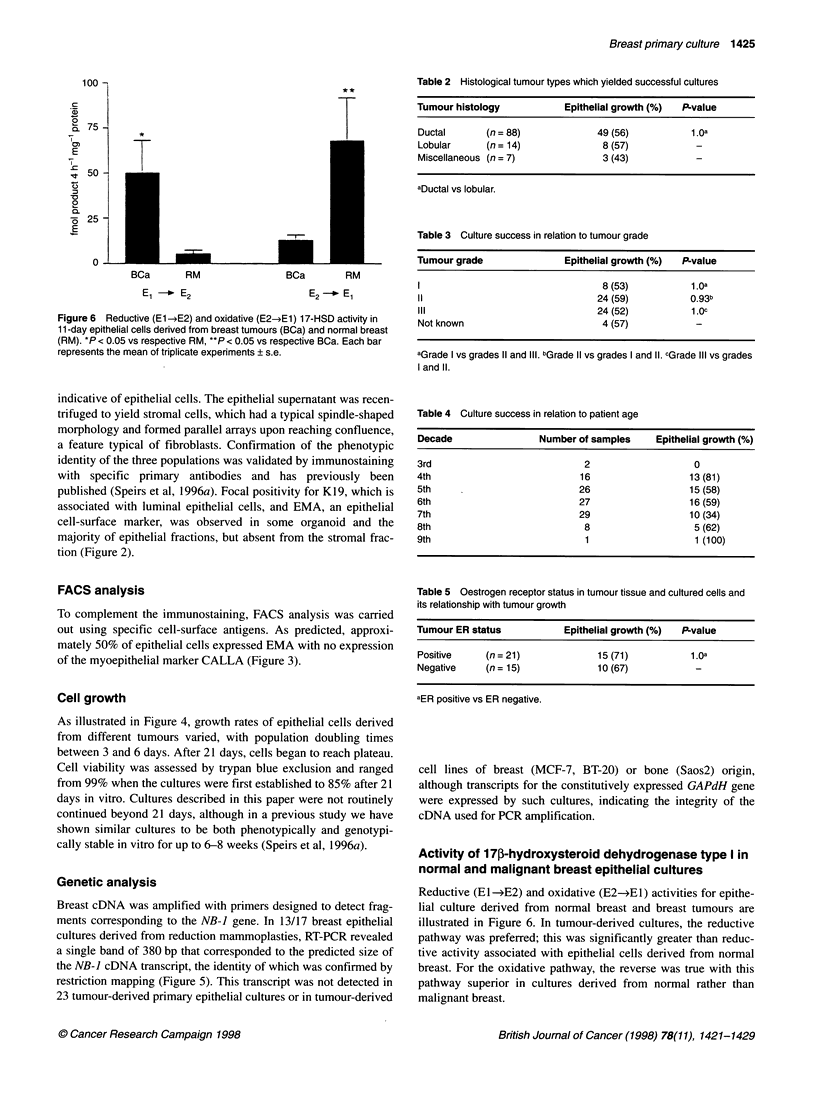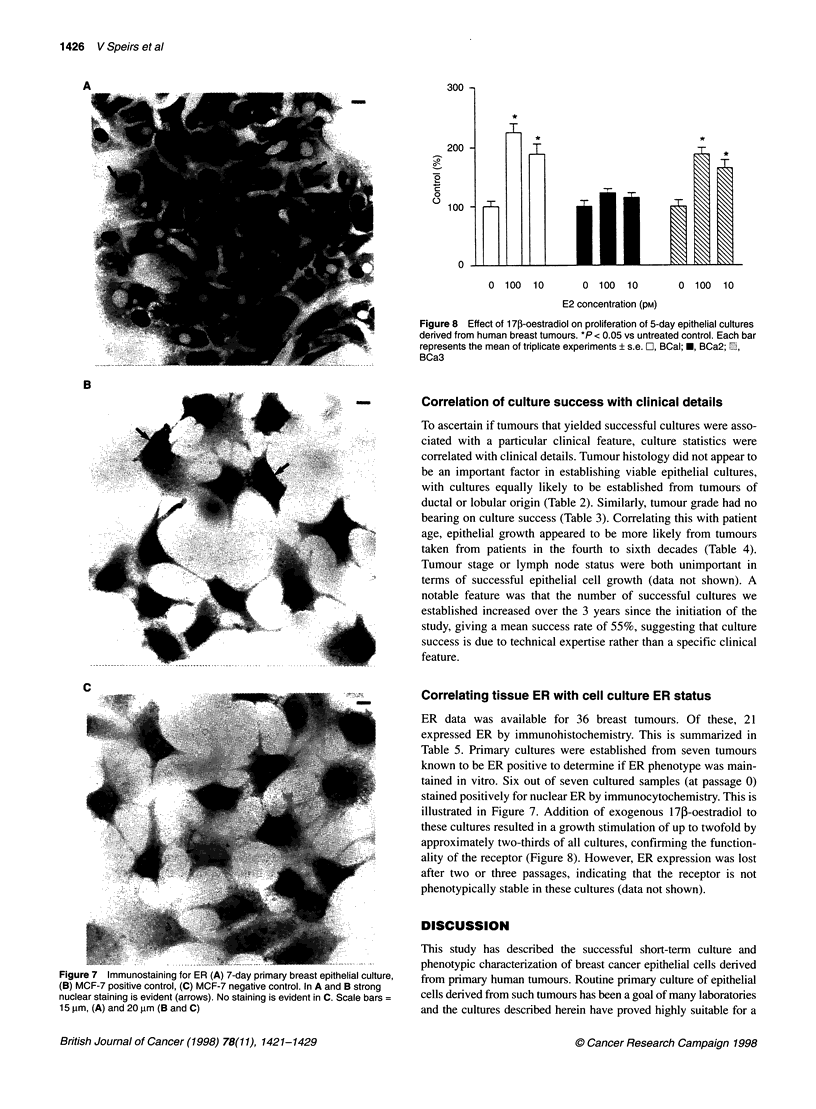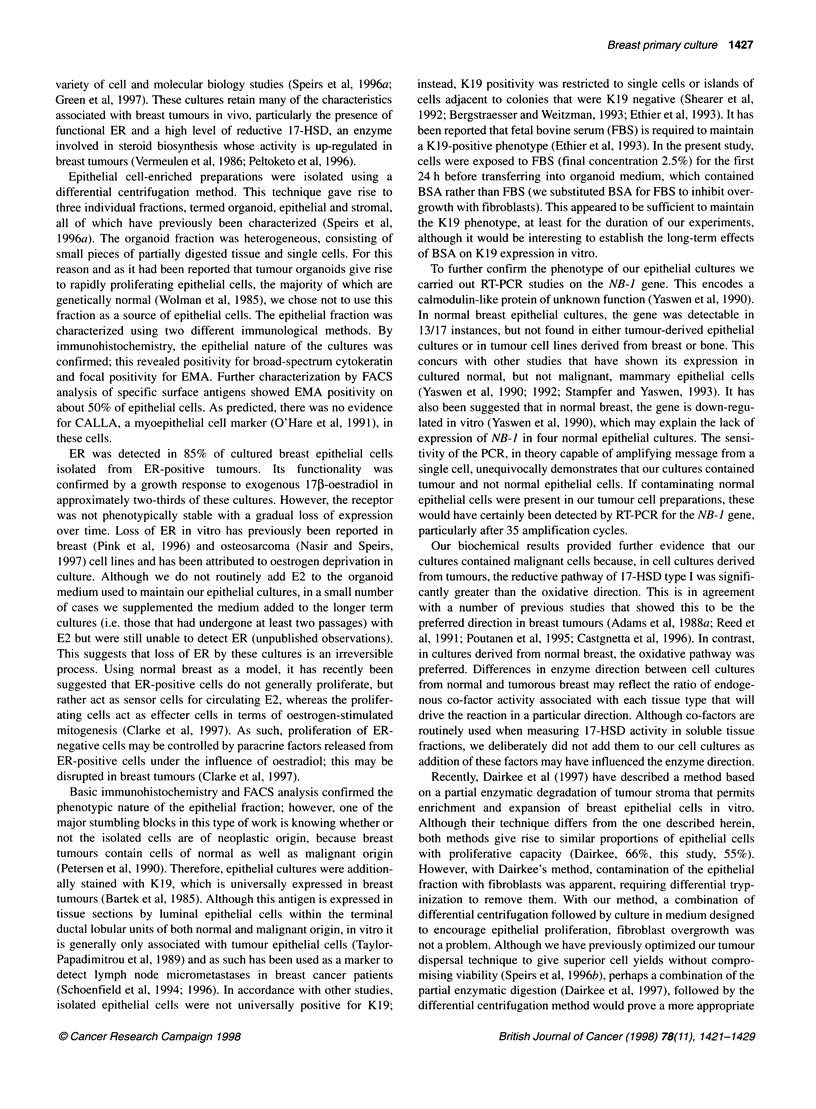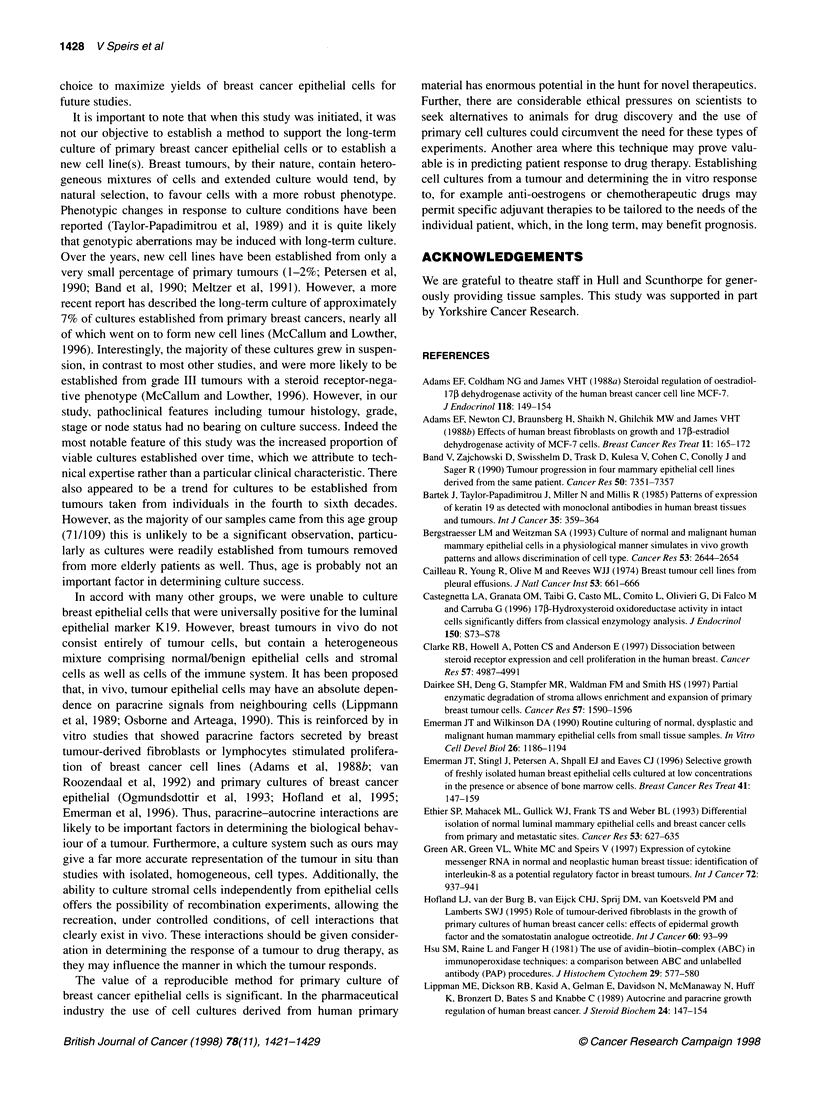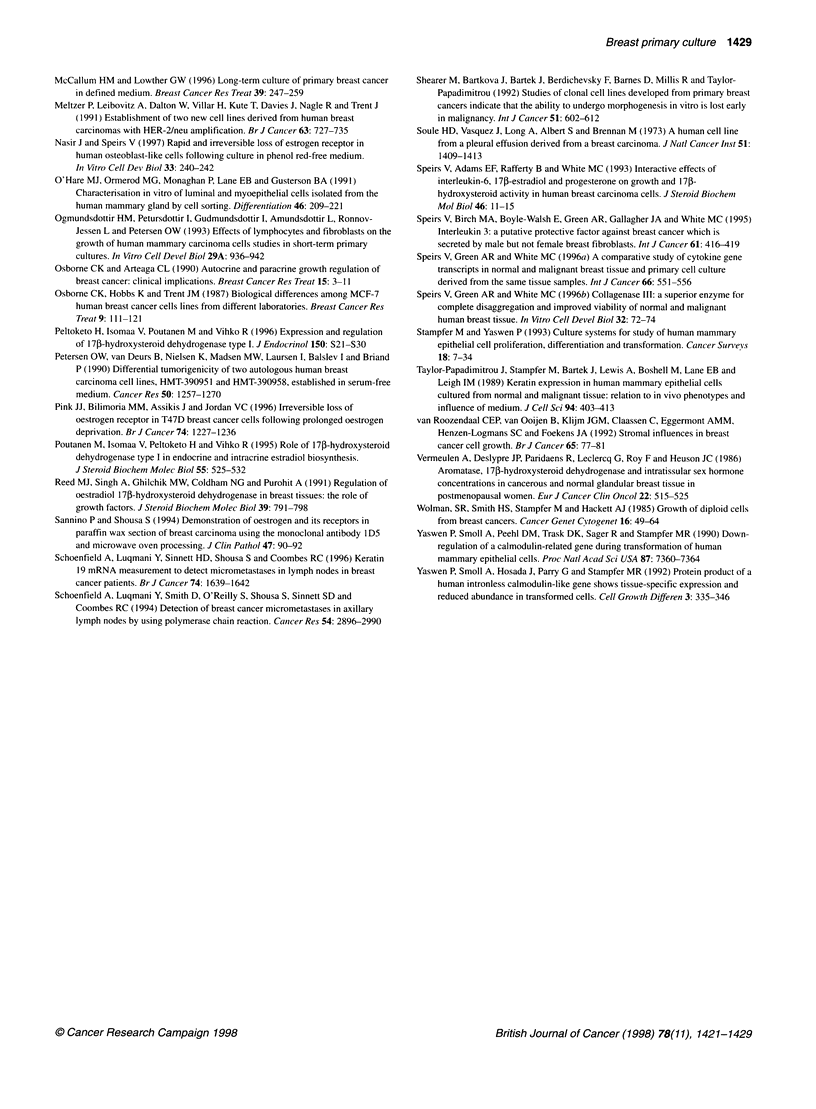# Short-term primary culture of epithelial cells derived from human breast tumours.

**DOI:** 10.1038/bjc.1998.702

**Published:** 1998-12

**Authors:** V. Speirs, A. R. Green, D. S. Walton, M. J. Kerin, J. N. Fox, P. J. Carleton, S. B. Desai, S. L. Atkin

**Affiliations:** Department of Medicine, University of Hull, UK.

## Abstract

**Images:**


British Journal of Cancer (1998) 78(11), 1421–1429
© 1998 Cancer Research Campaign

### Short-term primary culture of epithelial cells derived from human breast tumours

V Speirs[1], AR Green[1], DS Walton[1], MJ Kerin[2], JN Fox[2], PJ Carleton[2], SB Desai[3] and SL Atkin[1]

[1]Department of Medicine and [2]Academic Surgical Unit, University of Hull, Hull HU6 7RX; [3]Scunthorpe General Hospital, Scunthorpe DN15 7BH, UK

**Summary** As experimental models for breast cancer, most studies rely on established human breast cancer cell lines. However, many of these lines were established over 20 years ago, many from pleural effusions rather than the primary tumour, so the validity of using them as representative models is questionable. This paper describes our experiences, over a 3-year period, in establishing short-term epithelial-cell-enriched preparations from primary breast tumours based on differential centrifugation followed by culture in selective media. Epithelial cells were successfully cultured from 55% of samples, but culture success did not appear to be correlated with tumour histology, stage, grade or node status. Epithelial cell-enriched cultures were immunopositive for broad-spectrum cytokeratin and epithelial membrane antigen (EMA). Positivity for keratin 19 confirmed that the cultures contained tumour-derived cells, which additionally showed significantly higher activity of the reductive pathway of the steroid-converting enzyme 17β-hydroxysteroid dehydrogenase type I. That the cultures contained tumour and not normal epithelial cells was further substantiated by the complete absence of the calmodulin-like gene *NB-1* in tumour-derived cultures; this is only associated with normal breast epithelia. Eighty-five per cent of cultures established from oestrogen receptor (ER)-positive tumours expressed ER in vitro; this was functional in 66% of cultures, although ER-positive phenotype was gradually lost over time. In conclusion, epithelial cells can be isolated and maintained as short-term cultures from primary breast tumours irrespective of histopathological or clinical details, providing a model system with a greater biological and clinical relevance than breast cancer cell lines.

**Keywords:** breast; culture; epithelial; tumour

The majority of scientists and clinicians involved with breast cancer research use established breast cancer cell lines for in vitro studies, and a significant amount of useful information has been accumulated from these over the years. However, little consideration is given to the fact that many of these cell lines were originally described over 20 years ago and, with genetic drift, may bear very little resemblance to the primary tumour from which they were derived. This point is reinforced by the observation that conflicting results have often been reported from different laboratories in identical studies using the 'same' target cell (Osborne et al, 1987). Further, the most used in vitro model for breast cancer, the MCF-7 cell line, first described in 1973 (Soule et al, 1973), was actually derived from an aggressive metastatic pleural effusion, as is the case with most of the breast cancer cell lines currently in use today (Cailleau et al, 1974). Despite this, a database search of the literature over the past three decades using Medline (Win SPIRS version 2.0 on Silverplatter) and Bath Information Database Service (BIDS), revealed that the MCF-7 cell line still remains the model of choice as a research tool, with 97% of the published literature using this as a model for breast cancer and less than 3% of published studies using primary cultures derived from breast tumours.

The reasons for the continued use of cell lines as in vitro models are obvious, the prime one being their ease of use. Cell lines are well characterized homogeneous populations that (usually) give consistent and reproducible results from experiment to experiment.

*Received 23 December 1997*
*Revised 10 March 1998*
*Accepted 11 March 1998*

*Correspondence to:* V Speirs

They are also easily replaced if lost through contamination. Many of these lines express functional oestrogen and/or progesterone receptors and some have been characterized at the genetic level. Nevertheless, it has been a long-standing target of many laboratories to establish and maintain primary cultures, particularly of luminal epithelial cells, from which most breast cancers arise (Taylor-Papadimitrou et al, 1989), as a more representative in vitro model. However, these experiments have met with limited success, mainly because breast cancer epithelial cells can be difficult to establish, with slow doubling times. Furthermore, such cultures are often poorly characterized, with rapid overgrowth by fibroblasts frequently representing a considerable technical challenge.

The aim of this study was to establish a reproducible technique suitable for the short-term culture of human breast epithelial cells derived from breast tumours to provide sufficient quantities of cells for molecular and cell biology studies. Using a modification of a method based on differential centrifugation followed by culture in selective media (Emerson and Wilkinson, 1990; Speirs et al, 1996*a*), this report describes our experiences in establishing such cultures over a 3-year period and discusses their benefits over cell lines. The cultures have been characterized by a number of criteria including immunocytochemistry and flow cytometry with a range of primary antibodies, RT-PCR to detect the *NB-1* gene, a calmodulin-like protein of unknown function that is expressed in cultured normal, but not malignant, mammary epithelial cells (Yaswen et al, 1990; 1992; Stampfer and Yaswen, 1993) and the presence of increased reductive activity of the steroid-converting enzyme 17β-hydroxysteroid dehydrogenase type I (17-HSD), which is significantly up-regulated in breast tumours (Vermeulen et al, 1986; Peltoketo et al, 1996).

**Table 1** Pathoclinical details of breast tumours used in this study

| Clinical parameter | Numbers |
|---|---|
| Patients | |
| Total number in study | 109 |
| Age (years) | |
| Mean | 63 |
| Range | 32–90 |
| Tumour histology | |
| Duct origin | 88 |
| Lobule origin | 14 |
| Miscellaneous[a] | 7 |
| Tumour grade | |
| I | 15 |
| II | 41 |
| III | 46 |
| Not known | 7 |
| Lymph node status | |
| Positive | 58 |
| Negative | 48 |
| Not known | 3 |
| Tumour stage | |
| T1 | 25 |
| T2 | 73 |
| T3 | 5 |
| T4 | 1 |
| Not known | 5 |

[a]This group comprised one apocrine, one leiomyosarcoma, two medullary, two mixed lobular–ductal carcinoma and one tumour of ductal–mucoid origin.

**Figure 1** Schematic illustration of the cell separation procedure with representative phase contrast micrographs of the isolated cell fractions after 7 days in culture. Scale bar = 15 µm

### METHODS

### Tissue samples

Breast tumours were obtained from 109 patients undergoing surgery for the removal of a clinically confirmed breast lesion who presented sequentially. The mean age was 63 years (range 32–90). Clinicopathological details of the tumours are presented in Table 1.

### Cell isolation and culture

Upon receipt, tissue was washed extensively in phosphate-buffered saline (PBS) supplemented with 200 U of penicillin, 200 µg $ml^{-1}$ streptomycin and 5 µg $ml^{-1}$ fungizone (all Life Technologies, Paisley, UK), then minced finely and disaggregated for 18–20 in 0.1% collagenase type III (Life Technologies) as previously described (Speirs et al, 1996*b*). Digested tissue was removed from the incubator and shaken vigorously by hand to disaggregate any remaining large clumps. Three cell populations were then isolated using differential centrifugation (Emerson and Wilkinson, 1990). This is described in greater detail in the results section and illustrated schematically in Figure 1. For the first 24 h, cells from the organoid and epithelial fractions were plated in 75% organoid medium (OM) and 25% Dulbecco's modified Eagle medium (DMEM) containing 10% heat-inactivated fetal bovine serum (HIFBS) to promote cell attachment. OM consisted of DMEM supplemented with 100 U $ml^{-1}$ penicillin, 100 µg $ml^{-1}$ streptomycin, 2 mM glutamine, 10 mM Hepes, 0.075% bovine serum albumin (BSA), 10 ng $ml^{-1}$ cholera toxin (ICN Biomedicals, Oxon, UK), 0.5 µg $ml^{-1}$ hydrocortisone, 5 µg $ml^{-1}$ insulin and 5 ng $ml^{-1}$ epidermal growth factor (EGF) (all Sigma, Poole). The pH was 7.4. After 24 h media was removed and replaced with OM. Cells were maintained in this for the duration of the culture. Stromal cells were seeded in DMEM containing glutamine, penicillin, streptomycin and 10% HIFBS and maintained in this throughout.

### Cell characterization

### *Immunostaining*

To confirm the phenotypic identity of the isolated cells, immunostaining was carried out. Cultures were fixed with absolute methanol for 10 min at room temperature and immunostained using a Vectastain Quick kit (Vector Laboratories, Peterborough, UK). Fibroblasts were detected with vimentin (1:100; Sigma), whereas epithelial cells were detected with broad-spectrum cytokeratin (clone MNF116; 1:100; Dako, High Wycombe, UK), keratin 19 (K19, 1:100; Dako) and epithelial membrane antigen (EMA; 1:40; Dako). To detect ER-α, epithelial cultures were immunostained with ER antibody 1D5 (directed against the A/B domain of ER; Dako) purchased as an optimally prediluted solution, according to the manufacturer's instructions. Methanol-fixed cultures of the ER-positive breast carcinoma cell line, MCF-7, served as a positive control. As a negative control, cultures were incubated with negative control reagent provided with the primary antibody. This consisted of fetal calf serum in 0.05 M Tris-HCl, pH 7.6, containing carrier protein and 15 mM sodium azide. To determine if ER status in cell cultures correlated with that of the primary tumour from which they were derived, formalin-fixed paraffin-embedded

© Cancer Research Campaign 1998

**Figure 2** Eleven-day breast epithelial cultures. **A** K19-positive colony adjacent to cells that are K19 negative. (**B**) Focal positivity for EMA. Scale bars = 20 μm (**A**), 15 μm (**B**)

sections from breast tumours were also immunostained. Sections were dewaxed, rehydrated and, before immunostaining, sections were subjected to microwave enhancement as previously recommended for detection of ER (Sannino and Shousa, 1994). Positive immunostaining was detected using the ABC method (Hsu et al, 1981) with diaminobenzidine as a substrate.

### *Flow cytometry*

Flow cytometry analysis (FACS) was carried out on representative epithelial ($n$ = 8) and stromal ($n$ = 10) cultures. Cells were detached from the culture vessel by treatment with PBS/0.1% EDTA at 37°C, then incubated for 30 min at room temperature with antibodies against CALLA (which detects myoepithelial cells; O'Hare et al, 1991) or EMA. This was followed by a further 30 min incubation with FITC-conjugated secondary antibody (Becton Dickinson). For each sample a total of 10 000 cells were analysed (FACSCalibur, Becton Dickinson). Gates were set such that the negative control contained only 5% positive cells.

### *RT-PCR*

Total RNA was extracted from 23 tumour-derived epithelial cultures, 17 epithelial cultures derived from reduction mammoplasties, the breast cancer cell lines MCF-7 and BT-20 and the osteosarcoma cell line Saos2, and reverse transcribed to cDNA as previously described (Speirs et al, 1995). This was amplified with oligonucleotide primers designed to detect *NB-1*, a calmodulin-related gene expressed by most normal but not by malignant mammary epithelial cells (Yaswen et al, 1990; 1992; Stampfer and Yaswen, 1993). Primer sequences were as follows:

5′-TGT TTG ACA AGG ATG GGG AC-3′
5′-CAC ACG GAC AAA CTC CTC GT-3′

cDNA was amplified in a thermal cycler (Hybaid OmniGene; annealing temperature 55°C as previously described; Speirs et al, 1996*a*). Primers were designed to span introns so that signals from mRNA could be distinguished from any contaminating genomic DNA. To check for cDNA integrity, a fragment of the constitutively expressed glyceraldehyde phosphate dehydrogenase (GAPdH) gene was amplified and a reaction where sterile distilled water substituted for cDNA acted as a negative control. Product identity was confirmed by restriction mapping. An aliquot of 5 μl of PCR product was incubated with *Ban*I restriction enzyme (New England Biolabs) for 2 h at 37°C, which yielded two discrete fragments of 252 and 128 bp. Cleaved and uncleaved products were separated on a 2% agarose gel and visualized by ethidium bromide staining under UV illumination.

### Determination of cell number and viability

Direct cell counting and viability was performed on three cultures, as in general there were insufficient cells per tissue sample to sacrifice for cell counting only. To promote cell attachment, epithelial cells were plated at $2\times10^4$ cells per well in 24-well plates in 75% OM, 25% DMEM containing 10% HIFBS for 24 h before transferring to OM for the remainder of the experiment. Cells were counted every 5–6 days over a 21-day period with weekly media changes. Viability was determined by trypan blue exclusion.

### Measurement of 17β-hydroxysteroid dehydrogenase (17-HSD)

Primary epithelial cultures derived from breast tumours ($n$ = 11) and normal breast (reduction mammoplasties; $n$ = 10) were established

**Figure 3** Expression of EMA but not CALLA on 9-day breast tumour epithelial cells by FACS analysis. Peak a, irrelevant control antibody; peak b, EMA; peak c, CALLA. Fifty-two % of cells were positive for EMA

**Figure 4** Growth of breast cancer epithelial cells in vitro. Cells were seeded in 24-well plates and triplicate wells counted on days 6, 10, 15 and 21 post seeding. Each data point represents the mean of three wells ± s.e.;-■-, Bcal; -▲-, Bca2; -▼- BCa3

in six-well plates and allowed to reach 70% confluence. Oxidative and reductive 17-HSD type I activities were determined by measuring the ability of intact cell monolayers to convert reversibly exogenous [$^3$H]El to [$^3$H]E2 (Speirs et al, 1993). Cells were incubated with 2 nM of either $^3$[H]E1 (reductive pathway) or [$^3$H]E2 (oxidative pathway; both Amersham) in serum-free medium for 4 h at 37°C. Blanks were run in parallel. Following incubation, the medium was removed and added to glass tubes containing 5000 d.p.m. of either $^{14}$[C]E2 (reductive pathway) or [$^{14}$]CE1 (oxidative pathway; both Amersham) as a recovery label. Steroids were extracted with 4 ml of diethyl ether, evaporated to dryness and separated by thin-layer chromatography using a solvent system of dichloromethane–ethyl acetate (4:1). Product and recovery radioactivity were determined by liquid scintillation counting. Results were expressed as fmol of product mg$^{-1}$ protein 4 h$^{-1}$. Total protein was assessed by the BioRad protein assay (BioRad). All samples were assayed in duplicate.

**Figure 5** Representative agarose gel showing expression of NB-1 transcripts (380 bp; top) and the constitutively expressed GAPdH (326 bp; bottom). Top, lanes a, c, d and f show positive products for NB-1 obtained from 7-day cultures of normal breast epithelial cells. Age-matched tumour-derived cultures were negative (lanes b and e). Lane g, negative control; lane h, restriction mapped product giving fragments of 252 and 128 bp after digestion with *BanI*. Bottom, lanes a–f show positive GAPdH products for each sample. Lane g, negative control; lane h, restriction mapped product giving fragments of 228, 52 and 46 bp after digestion with *Hinf*. Note that the two small fragments migrated ahead of the dye front and are not visible on the photograph. L, 100 bp ladder (top and bottom)

### Statistical analysis

The chi-squared test was used to determine statistical significances for cell culture criteria and the unpaired Student's *t*-test was used for 17-HSD experiments.

### RESULTS

### Criteria used to ascertain culture success

Epithelial cultures were considered to be successful if they displayed the following features: (1) attachment and growth of phenotypically epithelial-like cells to the culture vessel after 3–5 days in vitro in defined medium; (2) continued growth, under these conditions, for up to 6–8 weeks with a doubling time of approximately 3–6 days, with no evidence of fibroblastic infiltration; (3) immunopositivity for broad-spectrum cytokeratin, clonal reactivity for K19 and EMA, with no expression of vimentin or CALLA; (4) lack of expression of the *NB-1* gene by RT-PCR; (5) preferential reductive 17-HSD activity in cell monolayers. These characteristics are described in greater detail below.

### Phenotypic identity

The differential centrifugation technique gave rise to cell populations from all three centrifugal fractions (Figure 1). The organoid fraction consisted predominantly of small fragments of partially digested tissue from which epithelial-like cells began to grow after 1–2 days in organoid medium. Epithelial cell-enriched cell pellets were obtained by recentrifuging the supernatant at higher speed, yielding mainly single cells, which, when cultured in organoid medium, displayed a characteristic cobblestone morphology

© Cancer Research Campaign 1998

**Figure 6** Reductive (E1→E2) and oxidative (E2→E1) 17-HSD activity in 11-day epithelial cells derived from breast tumours (BCa) and normal breast (RM). **P* < 0.05 vs respective RM, ***P* < 0.05 vs respective BCa. Each bar represents the mean of triplicate experiments ± s.e.

indicative of epithelial cells. The epithelial supernatant was recentrifuged to yield stromal cells, which had a typical spindle-shaped morphology and formed parallel arrays upon reaching confluence, a feature typical of fibroblasts. Confirmation of the phenotypic identity of the three populations was validated by immunostaining with specific primary antibodies and has previously been published (Speirs et al, 1996*a*). Focal positivity for K19, which is associated with luminal epithelial cells, and EMA, an epithelial cell-surface marker, was observed in some organoid and the majority of epithelial fractions, but absent from the stromal fraction (Figure 2).

### FACS analysis

To complement the immunostaining, FACS analysis was carried out using specific cell-surface antigens. As predicted, approximately 50% of epithelial cells expressed EMA with no expression of the myoepithelial marker CALLA (Figure 3).

### Cell growth

As illustrated in Figure 4, growth rates of epithelial cells derived from different tumours varied, with population doubling times between 3 and 6 days. After 21 days, cells began to reach plateau. Cell viability was assessed by trypan blue exclusion and ranged from 99% when the cultures were first established to 85% after 21 days in vitro. Cultures described in this paper were not routinely continued beyond 21 days, although in a previous study we have shown similar cultures to be both phenotypically and genotypically stable in vitro for up to 6–8 weeks (Speirs et al, 1996*a*).

### Genetic analysis

Breast cDNA was amplified with primers designed to detect fragments corresponding to the *NB-1* gene. In 13/17 breast epithelial cultures derived from reduction mammoplasties, RT-PCR revealed a single band of 380 bp that corresponded to the predicted size of the *NB-1* cDNA transcript, the identity of which was confirmed by restriction mapping (Figure 5). This transcript was not detected in 23 tumour-derived primary epithelial cultures or in tumour-derived

**Table 2** Histological tumour types which yielded successful cultures

| Tumour histology | | Epithelial growth (%) | *P*-value |
|---|---|---|---|
| Ductal | (*n* = 88) | 49 (56) | 1.0[a] |
| Lobular | (*n* = 14) | 8 (57) | – |
| Miscellaneous | (*n* = 7) | 3 (43) | – |

[a]Ductal vs lobular.

**Table 3** Culture success in relation to tumour grade

| Tumour grade | Epithelial growth (%) | *P*-value |
|---|---|---|
| I | 8 (53) | 1.0[a] |
| II | 24 (59) | 0.93[b] |
| III | 24 (52) | 1.0[c] |
| Not known | 4 (57) | – |

[a]Grade I vs grades II and III. [b]Grade II vs grades I and II. [c]Grade III vs grades I and II.

**Table 4** Culture success in relation to patient age

| Decade | Number of samples | Epithelial growth (%) |
|---|---|---|
| 3rd | 2 | 0 |
| 4th | 16 | 13 (81) |
| 5th | 26 | 15 (58) |
| 6th | 27 | 16 (59) |
| 7th | 29 | 10 (34) |
| 8th | 8 | 5 (62) |
| 9th | 1 | 1 (100) |

**Table 5** Oestrogen receptor status in tumour tissue and cultured cells and its relationship with tumour growth

| Tumour ER status | | Epithelial growth (%) | *P*-value |
|---|---|---|---|
| Positive | (*n* = 21) | 15 (71) | 1.0[a] |
| Negative | (*n* = 15) | 10 (67) | – |

[a]ER positive vs ER negative.

cell lines of breast (MCF-7, BT-20) or bone (Saos2) origin, although transcripts for the constitutively expressed *GAPdH* gene were expressed by such cultures, indicating the integrity of the cDNA used for PCR amplification.

### Activity of 17β-hydroxysteroid dehydrogenase type I in normal and malignant breast epithelial cultures

Reductive (E1→E2) and oxidative (E2→E1) activities for epithelial culture derived from normal breast and breast tumours are illustrated in Figure 6. In tumour-derived cultures, the reductive pathway was preferred; this was significantly greater than reductive activity associated with epithelial cells derived from normal breast. For the oxidative pathway, the reverse was true with this pathway superior in cultures derived from normal rather than malignant breast.

© Cancer Research Campaign 1998

Figure 7 Immunostaining for ER (A) 7-day primary breast epithelial culture, (B) MCF-7 positive control, (C) MCF-7 negative control. In A and B strong nuclear staining is evident (arrows). No staining is evident in C. Scale bars = 15 μm, (A) and 20 μm (B and C)

**Figure 8** Effect of 17β-oestradiol on proliferation of 5-day epithelial cultures derived from human breast tumours. *$P < 0.05$ vs untreated control. Each bar represents the mean of triplicate experiments ± s.e. □, BCal; ■, BCa2; ▧, BCa3

### Correlation of culture success with clinical details

To ascertain if tumours that yielded successful cultures were associated with a particular clinical feature, culture statistics were correlated with clinical details. Tumour histology did not appear to be an important factor in establishing viable epithelial cultures, with cultures equally likely to be established from tumours of ductal or lobular origin (Table 2). Similarly, tumour grade had no bearing on culture success (Table 3). Correlating this with patient age, epithelial growth appeared to be more likely from tumours taken from patients in the fourth to sixth decades (Table 4). Tumour stage or lymph node status were both unimportant in terms of successful epithelial cell growth (data not shown). A notable feature was that the number of successful cultures we established increased over the 3 years since the initiation of the study, giving a mean success rate of 55%, suggesting that culture success is due to technical expertise rather than a specific clinical feature.

### Correlating tissue ER with cell culture ER status

ER data was available for 36 breast tumours. Of these, 21 expressed ER by immunohistochemistry. This is summarized in Table 5. Primary cultures were established from seven tumours known to be ER positive to determine if ER phenotype was maintained in vitro. Six out of seven cultured samples (at passage 0) stained positively for nuclear ER by immunocytochemistry. This is illustrated in Figure 7. Addition of exogenous 17β-oestradiol to these cultures resulted in a growth stimulation of up to twofold by approximately two-thirds of all cultures, confirming the functionality of the receptor (Figure 8). However, ER expression was lost after two or three passages, indicating that the receptor is not phenotypically stable in these cultures (data not shown).

### DISCUSSION

This study has described the successful short-term culture and phenotypic characterization of breast cancer epithelial cells derived from primary human tumours. Routine primary culture of epithelial cells derived from such tumours has been a goal of many laboratories and the cultures described herein have proved highly suitable for a

© Cancer Research Campaign 1998

variety of cell and molecular biology studies (Speirs et al, 1996*a*; Green et al, 1997). These cultures retain many of the characteristics associated with breast tumours in vivo, particularly the presence of functional ER and a high level of reductive 17-HSD, an enzyme involved in steroid biosynthesis whose activity is up-regulated in breast tumours (Vermeulen et al, 1986; Peltoketo et al, 1996).

Epithelial cell-enriched preparations were isolated using a differential centrifugation method. This technique gave rise to three individual fractions, termed organoid, epithelial and stromal, all of which have previously been characterized (Speirs et al, 1996*a*). The organoid fraction was heterogeneous, consisting of small pieces of partially digested tissue and single cells. For this reason and as it had been reported that tumour organoids give rise to rapidly proliferating epithelial cells, the majority of which are genetically normal (Wolman et al, 1985), we chose not to use this fraction as a source of epithelial cells. The epithelial fraction was characterized using two different immunological methods. By immunohistochemistry, the epithelial nature of the cultures was confirmed; this revealed positivity for broad-spectrum cytokeratin and focal positivity for EMA. Further characterization by FACS analysis of specific surface antigens showed EMA positivity on about 50% of epithelial cells. As predicted, there was no evidence for CALLA, a myoepithelial cell marker (O'Hare et al, 1991), in these cells.

ER was detected in 85% of cultured breast epithelial cells isolated from ER-positive tumours. Its functionality was confirmed by a growth response to exogenous 17β-oestradiol in approximately two-thirds of these cultures. However, the receptor was not phenotypically stable with a gradual loss of expression over time. Loss of ER in vitro has previously been reported in breast (Pink et al, 1996) and osteosarcoma (Nasir and Speirs, 1997) cell lines and has been attributed to oestrogen deprivation in culture. Although we do not routinely add E2 to the organoid medium used to maintain our epithelial cultures, in a small number of cases we supplemented the medium added to the longer term cultures (i.e. those that had undergone at least two passages) with E2 but were still unable to detect ER (unpublished observations). This suggests that loss of ER by these cultures is an irreversible process. Using normal breast as a model, it has recently been suggested that ER-positive cells do not generally proliferate, but rather act as sensor cells for circulating E2, whereas the proliferating cells act as effecter cells in terms of oestrogen-stimulated mitogenesis (Clarke et al, 1997). As such, proliferation of ER-negative cells may be controlled by paracrine factors released from ER-positive cells under the influence of oestradiol; this may be disrupted in breast tumours (Clarke et al, 1997).

Basic immunohistochemistry and FACS analysis confirmed the phenotypic nature of the epithelial fraction; however, one of the major stumbling blocks in this type of work is knowing whether or not the isolated cells are of neoplastic origin, because breast tumours contain cells of normal as well as malignant origin (Petersen et al, 1990). Therefore, epithelial cultures were additionally stained with K19, which is universally expressed in breast tumours (Bartek et al, 1985). Although this antigen is expressed in tissue sections by luminal epithelial cells within the terminal ductal lobular units of both normal and malignant origin, in vitro it is generally only associated with tumour epithelial cells (Taylor-Papadimitrou et al, 1989) and as such has been used as a marker to detect lymph node micrometastases in breast cancer patients (Schoenfield et al, 1994; 1996). In accordance with other studies, isolated epithelial cells were not universally positive for K19; instead, K19 positivity was restricted to single cells or islands of cells adjacent to colonies that were K19 negative (Shearer et al, 1992; Bergstraesser and Weitzman, 1993; Ethier et al, 1993). It has been reported that fetal bovine serum (FBS) is required to maintain a K19-positive phenotype (Ethier et al, 1993). In the present study, cells were exposed to FBS (final concentration 2.5%) for the first 24 h before transferring into organoid medium, which contained BSA rather than FBS (we substituted BSA for FBS to inhibit overgrowth with fibroblasts). This appeared to be sufficient to maintain the K19 phenotype, at least for the duration of our experiments, although it would be interesting to establish the long-term effects of BSA on K19 expression in vitro.

To further confirm the phenotype of our epithelial cultures we carried out RT-PCR studies on the *NB-1* gene. This encodes a calmodulin-like protein of unknown function (Yaswen et al, 1990). In normal breast epithelial cultures, the gene was detectable in 13/17 instances, but not found in either tumour-derived epithelial cultures or in tumour cell lines derived from breast or bone. This concurs with other studies that have shown its expression in cultured normal, but not malignant, mammary epithelial cells (Yaswen et al, 1990; 1992; Stampfer and Yaswen, 1993). It has also been suggested that in normal breast, the gene is down-regulated in vitro (Yaswen et al, 1990), which may explain the lack of expression of *NB-1* in four normal epithelial cultures. The sensitivity of the PCR, in theory capable of amplifying message from a single cell, unequivocally demonstrates that our cultures contained tumour and not normal epithelial cells. If contaminating normal epithelial cells were present in our tumour cell preparations, these would have certainly been detected by RT-PCR for the *NB-1* gene, particularly after 35 amplification cycles.

Our biochemical results provided further evidence that our cultures contained malignant cells because, in cell cultures derived from tumours, the reductive pathway of 17-HSD type I was significantly greater than the oxidative direction. This is in agreement with a number of previous studies that showed this to be the preferred direction in breast tumours (Adams et al, 1988*a*; Reed et al, 1991; Poutanen et al, 1995; Castgnetta et al, 1996). In contrast, in cultures derived from normal breast, the oxidative pathway was preferred. Differences in enzyme direction between cell cultures from normal and tumorous breast may reflect the ratio of endogenous co-factor activity associated with each tissue type that will drive the reaction in a particular direction. Although co-factors are routinely used when measuring 17-HSD activity in soluble tissue fractions, we deliberately did not add them to our cell cultures as addition of these factors may have influenced the enzyme direction.

Recently, Dairkee et al (1997) have described a method based on a partial enzymatic degradation of tumour stroma that permits enrichment and expansion of breast epithelial cells in vitro. Although their technique differs from the one described herein, both methods give rise to similar proportions of epithelial cells with proliferative capacity (Dairkee, 66%, this study, 55%). However, with Dairkee's method, contamination of the epithelial fraction with fibroblasts was apparent, requiring differential trypinization to remove them. With our method, a combination of differential centrifugation followed by culture in medium designed to encourage epithelial proliferation, fibroblast overgrowth was not a problem. Although we have previously optimized our tumour dispersal technique to give superior cell yields without compromising viability (Speirs et al, 1996*b*), perhaps a combination of the partial enzymatic digestion (Dairkee et al, 1997), followed by the differential centrifugation method would prove a more appropriate

© Cancer Research Campaign 1998

choice to maximize yields of breast cancer epithelial cells for future studies.

It is important to note that when this study was initiated, it was not our objective to establish a method to support the long-term culture of primary breast cancer epithelial cells or to establish a new cell line(s). Breast tumours, by their nature, contain heterogeneous mixtures of cells and extended culture would tend, by natural selection, to favour cells with a more robust phenotype. Phenotypic changes in response to culture conditions have been reported (Taylor-Papadimitrou et al, 1989) and it is quite likely that genotypic aberrations may be induced with long-term culture. Over the years, new cell lines have been established from only a very small percentage of primary tumours (1–2%; Petersen et al, 1990; Band et al, 1990; Meltzer et al, 1991). However, a more recent report has described the long-term culture of approximately 7% of cultures established from primary breast cancers, nearly all of which went on to form new cell lines (McCallum and Lowther, 1996). Interestingly, the majority of these cultures grew in suspension, in contrast to most other studies, and were more likely to be established from grade III tumours with a steroid receptor-negative phenotype (McCallum and Lowther, 1996). However, in our study, pathoclinical features including tumour histology, grade, stage or node status had no bearing on culture success. Indeed the most notable feature of this study was the increased proportion of viable cultures established over time, which we attribute to technical expertise rather than a particular clinical characteristic. There also appeared to be a trend for cultures to be established from tumours taken from individuals in the fourth to sixth decades. However, as the majority of our samples came from this age group (71/109) this is unlikely to be a significant observation, particularly as cultures were readily established from tumours removed from more elderly patients as well. Thus, age is probably not an important factor in determining culture success.

In accord with many other groups, we were unable to culture breast epithelial cells that were universally positive for the luminal epithelial marker K19. However, breast tumours in vivo do not consist entirely of tumour cells, but contain a heterogeneous mixture comprising normal/benign epithelial cells and stromal cells as well as cells of the immune system. It has been proposed that, in vivo, tumour epithelial cells may have an absolute dependence on paracrine signals from neighbouring cells (Lippmann et al, 1989; Osborne and Arteaga, 1990). This is reinforced by in vitro studies that showed paracrine factors secreted by breast tumour-derived fibroblasts or lymphocytes stimulated proliferation of breast cancer cell lines (Adams et al, 1988*b*; van Roozendaal et al, 1992) and primary cultures of breast cancer epithelial (Ogmundsdottir et al, 1993; Hofland et al, 1995; Emerman et al, 1996). Thus, paracrine–autocrine interactions are likely to be important factors in determining the biological behaviour of a tumour. Furthermore, a culture system such as ours may give a far more accurate representation of the tumour in situ than studies with isolated, homogeneous, cell types. Additionally, the ability to culture stromal cells independently from epithelial cells offers the possibility of recombination experiments, allowing the recreation, under controlled conditions, of cell interactions that clearly exist in vivo. These interactions should be given consideration in determining the response of a tumour to drug therapy, as they may influence the manner in which the tumour responds.

The value of a reproducible method for primary culture of breast cancer epithelial cells is significant. In the pharmaceutical industry the use of cell cultures derived from human primary material has enormous potential in the hunt for novel therapeutics. Further, there are considerable ethical pressures on scientists to seek alternatives to animals for drug discovery and the use of primary cell cultures could circumvent the need for these types of experiments. Another area where this technique may prove valuable is in predicting patient response to drug therapy. Establishing cell cultures from a tumour and determining the in vitro response to, for example anti-oestrogens or chemotherapeutic drugs may permit specific adjuvant therapies to be tailored to the needs of the individual patient, which, in the long term, may benefit prognosis.

### ACKNOWLEDGEMENTS

We are grateful to theatre staff in Hull and Scunthorpe for generously providing tissue samples. This study was supported in part by Yorkshire Cancer Research.

### REFERENCES

Adams EF, Coldham NG and James VHT (1988*a*) Steroidal regulation of oestradiol-17β dehydrogenase activity of the human breast cancer cell line MCF-7. *J Endocrinol* **118**: 149–154

Adams EF, Newton CJ, Braunsberg H, Shaikh N, Ghilchik MW and James VHT (1988*b*) Effects of human breast fibroblasts on growth and 17β-estradiol dehydrogenase activity of MCF-7 cells. *Breast Cancer Res Treat* **11**: 165–172

Band V, Zajchowski D, Swisshelm D, Trask D, Kulesa V, Cohen C, Conolly J and Sager R (1990) Tumour progression in four mammary epithelial cell lines derived from the same patient. *Cancer Res* **50**: 7351–7357

Bartek J, Taylor-Papadimitrou J, Miller N and Millis R (1985) Patterns of expression of keratin 19 as detected with monoclonal antibodies in human breast tissues and tumours. *Int J Cancer* **35**: 359–364

Bergstraesser LM and Weitzman SA (1993) Culture of normal and malignant human mammary epithelial cells in a physiological manner simulates in vivo growth patterns and allows discrimination of cell type. *Cancer Res* **53**: 2644–2654

Cailleau R, Young R, Olive M and Reeves WJJ (1974) Breast tumour cell lines from pleural effusions. *J Natl Cancer Inst* **53**: 661–666

Castegnetta LA, Granata OM, Taibi G, Casto ML, Comito L, Olivieri G, Di Falco M and Carruba G (1996) 17β-Hydroxysteroid oxidoreductase activity in intact cells significantly differs from classical enzymology analysis. *J Endocrinol* **150**: S73–S78

Clarke RB, Howell A, Potten CS and Anderson E (1997) Dissociation between steroid receptor expression and cell proliferation in the human breast. *Cancer Res* **57**: 4987–4991

Dairkee SH, Deng G, Stampfer MR, Waldman FM and Smith HS (1997) Partial enzymatic degradation of stroma allows enrichment and expansion of primary breast tumour cells. *Cancer Res* **57**: 1590–1596

Emerman JT and Wilkinson DA (1990) Routine culturing of normal, dysplastic and malignant human mammary epithelial cells from small tissue samples. *In Vitro Cell Devel Biol* **26**: 1186–1194

Emerman JT, Stingl J, Petersen A, Shpall EJ and Eaves CJ (1996) Selective growth of freshly isolated human breast epithelial cells cultured at low concentrations in the presence or absence of bone marrow cells. *Breast Cancer Res Treat* **41**: 147–159

Ethier SP, Mahacek ML, Gullick WJ, Frank TS and Weber BL (1993) Differential isolation of normal luminal mammary epithelial cells and breast cancer cells from primary and metastatic sites. *Cancer Res* **53**: 627–635

Green AR, Green VL, White MC and Speirs V (1997) Expression of cytokine messenger RNA in normal and neoplastic human breast tissue: identification of interleukin-8 as a potential regulatory factor in breast tumours. *Int J Cancer* **72**: 937–941

Hofland LJ, van der Burg B, van Eijck CHJ, Sprij DM, van Koetsveld PM and Lamberts SWJ (1995) Role of tumour-derived fibroblasts in the growth of primary cultures of human breast cancer cells: effects of epidermal growth factor and the somatostatin analogue octreotide. *Int J Cancer* **60**: 93–99

Hsu SM, Raine L and Fanger H (1981) The use of avidin–biotin–complex (ABC) in immunoperoxidase techniques: a comparison between ABC and unlabelled antibody (PAP) procedures. *J Histochem Cytochem* **29**: 577–580

Lippman ME, Dickson RB, Kasid A, Gelman E, Davidson N, McManaway N, Huff K, Bronzert D, Bates S and Knabbe C (1989) Autocrine and paracrine growth regulation of human breast cancer. *J Steroid Biochem* **24**: 147–154

© Cancer Research Campaign 1998

McCallum HM and Lowther GW (1996) Long-term culture of primary breast cancer in defined medium. *Breast Cancer Res Treat* **39**: 247–259

Meltzer P, Leibovitz A, Dalton W, Villar H, Kute T, Davies J, Nagle R and Trent J (1991) Establishment of two new cell lines derived from human breast carcinomas with HER-2/neu amplification. *Br J Cancer* **63**: 727–735

Nasir J and Speirs V (1997) Rapid and irreversible loss of estrogen receptor in human osteoblast-like cells following culture in phenol red-free medium. *In Vitro Cell Dev Biol* **33**: 240–242

O'Hare MJ, Ormerod MG, Monaghan P, Lane EB and Gusterson BA (1991) Characterisation in vitro of luminal and myoepithelial cells isolated from the human mammary gland by cell sorting. *Differentiation* **46**: 209–221

Ogmundsdottir HM, Petursdottir I, Gudmundsdottir I, Amundsdottir L, Ronnov-Jessen L and Petersen OW (1993) Effects of lymphocytes and fibroblasts on the growth of human mammary carcinoma cells studies in short-term primary cultures. *In Vitro Cell Devel Biol* **29A**: 936–942

Osborne CK and Arteaga CL (1990) Autocrine and paracrine growth regulation of breast cancer: clinical implications. *Breast Cancer Res Treat* **15**: 3–11

Osborne CK, Hobbs K and Trent JM (1987) Biological differences among MCF-7 human breast cancer cells lines from different laboratories. *Breast Cancer Res Treat* **9**: 111–121

Peltoketo H, Isomaa V, Poutanen M and Vihko R (1996) Expression and regulation of 17β-hydroxysteroid dehydrogenase type I. *J Endocrinol* **150**: S21–S30

Petersen OW, van Deurs B, Nielsen K, Madsen MW, Laursen I, Balslev I and Briand P (1990) Differential tumorigenicity of two autologous human breast carcinoma cell lines, HMT-390951 and HMT-390958, established in serum-free medium. *Cancer Res* **50**: 1257–1270

Pink JJ, Bilimoria MM, Assikis J and Jordan VC (1996) Irreversible loss of oestrogen receptor in T47D breast cancer cells following prolonged oestrogen deprivation. *Br J Cancer* **74**: 1227–1236

Poutanen M, Isomaa V, Peltoketo H and Vihko R (1995) Role of 17β-hydroxysteroid dehydrogenase type I in endocrine and intracrine estradiol biosynthesis. *J Steroid Biochem Molec Biol* **55**: 525–532

Reed MJ, Singh A, Ghilchik MW, Coldham NG and Purohit A (1991) Regulation of oestradiol 17β-hydroxysteroid dehydrogenase in breast tissues: the role of growth factors. *J Steroid Biochem Molec Biol* **39**: 791–798

Sannino P and Shousa S (1994) Demonstration of oestrogen and its receptors in paraffin wax section of breast carcinoma using the monoclonal antibody 1D5 and microwave oven processing. *J Clin Pathol* **47**: 90–92

Schoenfield A, Luqmani Y, Sinnett HD, Shousa S and Coombes RC (1996) Keratin 19 mRNA measurement to detect micrometastases in lymph nodes in breast cancer patients. *Br J Cancer* **74**: 1639–1642

Schoenfield A, Luqmani Y, Smith D, O'Reilly S, Shousa S, Sinnett SD and Coombes RC (1994) Detection of breast cancer micrometastases in axillary lymph nodes by using polymerase chain reaction. *Cancer Res* **54**: 2896–2990

Shearer M, Bartkova J, Bartek J, Berdichevsky F, Barnes D, Millis R and Taylor-Papadimitrou (1992) Studies of clonal cell lines developed from primary breast cancers indicate that the ability to undergo morphogenesis in vitro is lost early in malignancy. *Int J Cancer* **51**: 602–612

Soule HD, Vasquez J, Long A, Albert S and Brennan M (1973) A human cell line from a pleural effusion derived from a breast carcinoma. *J Natl Cancer Inst* **51**: 1409–1413

Speirs V, Adams EF, Rafferty B and White MC (1993) Interactive effects of interleukin-6, 17β-estradiol and progesterone on growth and 17β-hydroxysteroid activity in human breast carcinoma cells. *J Steroid Biochem Mol Biol* **46**: 11–15

Speirs V, Birch MA, Boyle-Walsh E, Green AR, Gallagher JA and White MC (1995) Interleukin 3: a putative protective factor against breast cancer which is secreted by male but not female breast fibroblasts. *Int J Cancer* **61**: 416–419

Speirs V, Green AR and White MC (1996*a*) A comparative study of cytokine gene transcripts in normal and malignant breast tissue and primary cell culture derived from the same tissue samples. *Int J Cancer* **66**: 551–556

Speirs V, Green AR and White MC (1996*b*) Collagenase III: a superior enzyme for complete disaggregation and improved viability of normal and malignant human breast tissue. *In Vitro Cell Devel Biol* **32**: 72–74

Stampfer M and Yaswen P (1993) Culture systems for study of human mammary epithelial cell proliferation, differentiation and transformation. *Cancer Surveys* **18**: 7–34

Taylor-Papadimitrou J, Stampfer M, Bartek J, Lewis A, Boshell M, Lane EB and Leigh IM (1989) Keratin expression in human mammary epithelial cells cultured from normal and malignant tissue: relation to in vivo phenotypes and influence of medium. *J Cell Sci* **94**: 403–413

van Roozendaal CEP, van Ooijen B, Klijm JGM, Claassen C, Eggermont AMM, Henzen-Logmans SC and Foekens JA (1992) Stromal influences in breast cancer cell growth. *Br J Cancer* **65**: 77–81

Vermeulen A, Deslypre JP, Paridaens R, Leclercq G, Roy F and Heuson JC (1986) Aromatase, 17β-hydroxysteroid dehydrogenase and intratissular sex hormone concentrations in cancerous and normal glandular breast tissue in postmenopausal women. *Eur J Cancer Clin Oncol* **22**: 515–525

Wolman, SR, Smith HS, Stampfer M and Hackett AJ (1985) Growth of diploid cells from breast cancers. *Cancer Genet Cytogenet* **16**: 49–64

Yaswen P, Smoll A, Peehl DM, Trask DK, Sager R and Stampfer MR (1990) Down-regulation of a calmodulin-related gene during transformation of human mammary epithelial cells. *Proc Natl Acad Sci USA* **87**: 7360–7364

Yaswen P, Smoll A, Hosada J, Parry G and Stampfer MR (1992) Protein product of a human intronless calmodulin-like gene shows tissue-specific expression and reduced abundance in transformed cells. *Cell Growth Differen* **3**: 335–346

© Cancer Research Campaign 1998